# The metagenome of bromeliads phytotelma in Puerto Rico

**DOI:** 10.1016/j.dib.2017.10.065

**Published:** 2017-11-01

**Authors:** Kiara M. Rodriguez-Nuñez, Jesie M. Rullan-Cardec, Carlos Rios-Velazquez

**Affiliations:** Biology Department, University of Puerto Rico at Mayagüez, Call Box 9000, Mayagüez, PR 00681–9000, United States

**Keywords:** Bromeliads, Phytotelma, DNA, Metagenome, Characterization, Illumina

## Abstract

Bromeliads tank water or phytotelma is an eutrophic microenvironment where microorganisms have evolved to resist sudden changes in pH and nutritional competition. Metagenomics studies have been poorly studied in bromeliads and environmental DNA (eDNA) characterization for its microenvironment is deficient in Puerto Rico. Therefore, the data represents the microbial communities inhabiting bromeliads phytotelma. eDNA was extracted using Metagenomic DNA Isolation Kit for Water. Next-Generation-Sequencing technology (Illumina MiSeq) was used for sequencing the isolated eDNA. This data provides an insight about diversity and functional depiction of microorganisms inhabiting bromeliads phytotelma. The data of this metagenome is available in the BioSample Submission Portal as Bioproject PRJNA39461 and Sequence Read Archive (SRA) accession number SRP114300. MG-RAST metagenomic analysis server is located under the study ID **mgp79812**.

**Specifications Table**

**Table:** 

Subject area	*Biology*
More specific subject area	*Metagenomics*
Type of data	*Fasta Q file*
How data was acquired	*Illumina MiSeq*
Data format	*Raw data*
Experimental factors	*Environmental samples*
Experimental features	*DNA extraction and sequencing of water from bromeliad phytotelmata*
Data source location	*Mayagüez, Puerto Rico* (*18° 12′32.7′′N 67°08′ 27.8′′W*)
Data accessibility	*The data of this metagenome is available in the NCBI BioSample Submission Portal as Bioproject PRJNA394613 and SRA accession number SRP114300. Study ID in the MG-RAST metagenomic analysis server is mgp79812* (http://metagenomics.anl.gov/mgmain.html?mgpage=project&project=mgp79812).

**Value of the data**

• This data represents the first metagenomic sequences of eDNA isolated from bromeliads phytotelma in Puerto Rico, to our knowledge.
• Bromeliad phytotelmata metagenomics provides valuable information for the study of microbial composition and its ecological roles of this poor studied environment.
• In addition, the data provided can be used for the study of bromeliad phytotelmata as bioprospect reservoir for biotechnological, industrial and biomedical applications.
• Unlike previous studies in which metagenomics libraries have been done from the detritus material eDNA [1], [2], the metagenome presented here is primarily from bromeliads phytotelma or tank water eDNA.
• This metagenome is valuable for the study and comparison of microbial communities among different types of phytotelmata.

## 1 Data

**Fig. 1:**
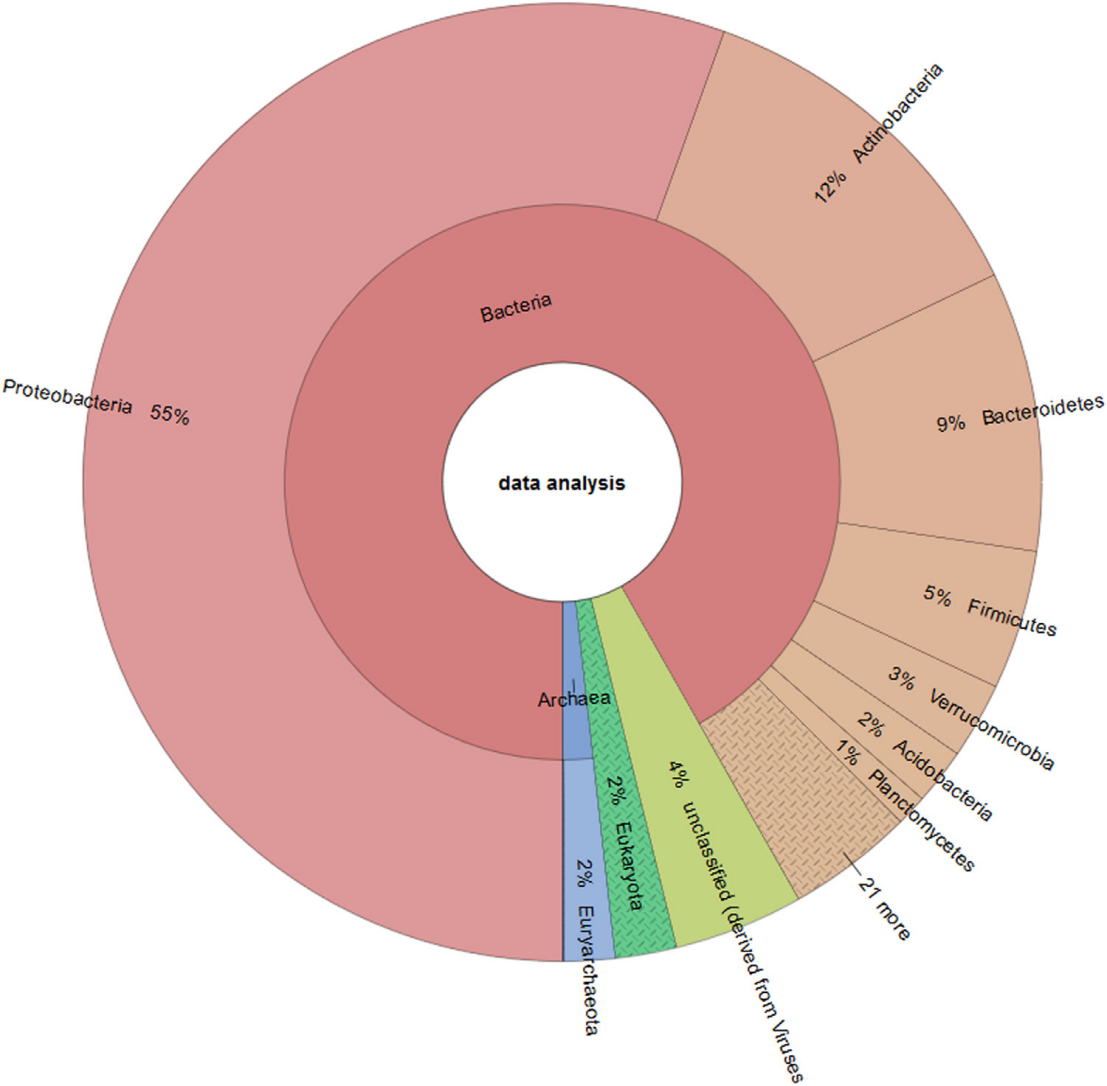
Analyzed sequences showed the phyla percentage of the bromeliads phytotelma microenvironment. The analyzed sequences displayed that microbial communities inhabiting were Proteobacteria (55%), Actinobacteria (12%) and other phyla (33%). Bromeliads phytotelma suggests an ubiquitous environment with a possible biotechnological potential.

Metagenomics studies have overcome the limitations of culture dependent microorganism studies. Microbial diversity, enzymatic activities and ecological roles of microorganisms in the desired environment can be studied without the necessity of microorganism isolation. Metagenomics have been used for the study of different aquatic environments such as lakes [3], oceans [4], rivers [5], hydrothermal vents [6] and hot springs [7]. New species of non-cultivable microorganisms, enzymes of biomedical and biotechnological value, and new metabolic pathways have been elucidated using metagenomics. While the applications of metagenomic studies are vast, bromeliad phytotelmata microbial and functional diversity have not been extensively explored using this technique. Bromeliad (Family *Bromeliaceae*) phytotelmata is an aquatic microenvironment formed by the accumulation of water between the axils of the leaves. It provides acidic and anaerobic conditions with high content of organic matter and nitrogen to the microbial communities [8]. To our understanding, there are only two studies about bromeliad phytotelmata using metagenomics, both in Brazil [1], [2]. The studies described microbial diversity in this microenvironment as unique when compared to other aquatic habitats and Bromeliad species. This data describes microbial communities inhabiting bromeliads phytotelma, its ecological roles and applications which are not well studied. A total of 51 phyla were identified, of which *Proteobacteria* (55%), *Actinobacteria* (12%) and Bacteroidetes (9%) were the most abundant, as shown in Fig. 1.

## 2 Experimental design, materials and methods

### 2.1 Sample collection

Sampled bromeliads, *Aechmea bromeliifolia* and *Aechmea nudicaulis*, were selected randomly from Celis Building garden in the University of Puerto Rico Mayaguez Campus (18° 12′32.7′′N 67°08′ 27.8′′W). In a sterile bottle, content from the axils of the center leaves (primarily accumulated water, and seldom detritus) of 16 bromeliads were collected for a total volume of approximately 400 mL (25 mL from each bromeliad). Temperature, salinity and pH of the sample was 20 °C, 0% and 5.69% respectively.

### 2.2 DNA extraction and metagenome sequencing

To extract the DNA from the phytotelmata microenvironment (pmDNA) the protocol of Metagenomic DNA Isolation Kit from Water (Epicentre Cat No. MGD08420) was followed for 200 mL of sample. After extracting the pmDNA, it was precipitated by adding 1/10 volume of 3 M sodium acetate, 2 volumes of cold absolute alcohol, followed by an hour of incubation in − 20 °C. The sample was centrifuged for 20 min at 15,000×*g*. Supernatant was discarded and the pellet was centrifuged for 5 minutes at 15,000×*g*. The pellet was washed with 70% absolute ethanol and re-suspended in TE buffer. The pmDNA was sent to MR DNA (http://www.mrdnalab.com) for sequencing. The initial DNA concentration was measured using Qubit® dsDNA HS Assay Kit (Life Technologies). 50 ng of pmDNA was used to prepare the library using Nextera DNA Sample preparation kit (Illumina) following manufacturer instructions. The pmDNA was fragmented and adapter sequences were added. Final DNA concentration in the libraries was 8.86 ng/µL with an average library size of 649 bp determined with Agilent 2100 Bioanalyzer (Agilent Technologies). The library was diluted to 12 pM and a 600 cycle v3 Reagent Kit was used for sequencing on the MiSeq(Illumina).
