# Supplementary material for: The metagenome of bromeliads phytotelma in Puerto Rico
Source: Data Brief. 2017 Nov 1;16:19–22. doi: 10.1016/j.dib.2017.10.065 (PMC5686464; doi:10.1016/j.dib.2017.10.065)
Supplement: Supplementary file 1 — Transparency document [file mmc1.docx]

October 11, 2017

Conflict of Interest form

To whom it may concern,

The authors confirm that there are no known conflicts of interest associated with this publication and there has been no significant financial support for this work that could have influenced its outcome.

Sincerely,

Kiara M. Rodríguez Núñez
